# Comparative performance of contact plate metod and swab method for surface microbial contamination on medical fabrics

**DOI:** 10.1186/s12879-024-09416-8

**Published:** 2024-05-27

**Authors:** Feng Chen, Yaru Li, Wanqiu Wang, Juan Li, Dong Wang, Xiaxia Sun, Yaping Peng, Jianjun Deng

**Affiliations:** 1 Department of Nosocomial Infection Management, West China Second University Hospital, Sichuan University, No. 20, Section 3, Renmin South Road,Wuhou District, Chengdu, Sichuan Province 610041 PR China; 2 Key Laboratory of Birth Defects and Related Diseases of Women and Children, Sichuan University, Ministry of Education, No. 20, Section 3, Renmin South Road,Wuhou District, Chengdu, Sichuan Province 610041 PR China; 3 Department of medical administration, West China Second University Hospital, Sichuan University, No. 20, Section 3, Renmin South Road,Wuhou District, Chengdu, Sichuan Province 610041 PR China

**Keywords:** Contact plate method, Hospital-acquired infection, Linear mixed-effects model, Medical fabrics

## Abstract

**Background:**

The contact plate method is widely accepted and used in various fields where hygiene and contamination levels are crucial. Evidence regarding the applicability of the contact plate method for sampling fabric microbial contamination levels in real medical environments was limited. This study aimed to assess the applicability of the contact plate method for detecting microbial contamination on medical fabrics in a real healthcare environment, thereby providing a benchmark for fabric microbial sampling methods.

**Methods:**

In a level three obstetrics ward of a hospital, twenty-four privacy curtains adjacent to patient beds were selected for this study. The contact plate and swab method were used to collect microbial samples from the privacy curtains on the 1st, 7th, 14th, and 28th days after they were hung. The total colony count on each privacy curtain surface was calculated, and microbial identification was performed.

**Results:**

After excluding the effects of time, room type, and curtain location on the detected microbial load, the linear mixed-effects model analysis showed that contact plate method yielded lower colony counts compared to swab method (*P* < 0.001). However, the contact plate method isolated more microbial species than swab method (*P* < 0.001). 291 pathogenic strains were isolated using the contact plate method and 133 pathogenic strains were isolated via the swab method. There was no difference between the two sampling methods in the detection of gram-negative bacteria (*P* = 0.089). Furthermore, the microbial load on curtains in double-occupancy rooms was lower than those in triple-occupancy rooms (*P* = 0.021), and the microbial load on curtains near windows was lower than that near doors (*P* = 0.004).

**Conclusion:**

Contact plate method is superior to swab method in strain isolation. Swab method is more suitable for evaluating the bacterial contamination of fabrics.

## Background

Hospital-acquired infection (HAI) is one of the most common adverse events during medical treatment. According to the 2022 WHO Global Report on Infection Prevention and Control in Emergency Health Facilities, seven patients in high-income countries and 15 patients in middle and low-income countries experience at least one HAI during their hospital stay for every 100 patients in acute-care hospitals [1, 2]. In middle and low-income countries, the risk of hospital-acquired infections (HAIs) in intensive care patients is 2–20 times higher than in high-income countries, particularly among newborns [2, 3]. HAIs can significantly impact patient outcomes and even endanger patients’ lives [4]. In Europe, approximately 37,000 people die annually from HAIs, and 25,000 people die of infections caused by multidrug-resistant organisms [5]. Researchers showed that 40–60% of HAIs are caused by endogenous flora, 20–40% are due to contaminated hands of healthcare workers, and 20% may result from environmental contamination [6].

As a potential reservoir for pathogens, the hospital surface environment is critical to infection risk. According to reports, when terminal cleaning and disinfection of hospital rooms are inadequate, the likelihood of a subsequent patient acquiring Methicillin-resistant Staphylococcus aureus (MRSA) or Vancomycinresistant Enterococcus (VRE) colonization or infection increases by 40%∼60% if the previous occupant had MRSA or VRE [7]. As frequently touched surfaces within hospital rooms, patient privacy curtains beside the bed serve as a reservoir for pathogens. Investigations have suggested that hospital textiles may also be a source of infections caused by streptococci [8], Bacillus cereus [9], staphylococci [10], and enteric bacteria [11]. Furthermore, studies have linked curtain contamination to certain outbreaks of HAIs. Curtains were identified as the primary host for carbapenem-resistant Acinetobacter baumannii in an intensive care unit (ICU) based on an investigation conducted in 2002 [12]. Another study found that curtains could serve as a source for cross-contamination of Group A Streptococcus in an ear, nose, and throat ward [13].

Currently, microbial sampling of the patient’s privacy curtain is not a routine environmental health monitoring project; therefore, its contamination level is unclear. Surface bacterial contamination sampling is critical for routine environmental monitoring, evaluating cleanliness, and investigating epidemics [14]. Depending on the condition of the fabric after sampling, fabric sampling methods can be categorized into two types: destructive testing and non-destructive testing [15]. The cotton swab is the most common sampling method for hospital environmental surfaces. However, the guidelines issued by the United Kingdom Department of Public Health recommend swabbing and contact plates when assessing the effectiveness of fabric cleaning and disinfection [5]. This method, which was originally proposed by Hall and Hartnett [16], is used for direct contact sampling of object surfaces. The contact plate method is widely accepted and used in various fields where hygiene and contamination levels are crucial, particularly in hospitals and food production facilities.

Compared to the swabbing technique, the contact plate sampling method can better recover infectious bacteria from the environment [17, 18]. In studies on the recovery of multidrug-resistant bacteria, the contact plate method has shown higher efficiency than the cotton swab method. The contact plate method is simple to perform, convenient for transportation, and suitable for sampling bacterial contamination on flat surfaces. Although the swabbing technique is widely used, it lacks the standardized conditions necessary for reproducible results [15]. Currently, studies on the recovery of bacteria using both swab and contact plate sampling methods are primarily conducted in controlled laboratory environments with single or multiple bacterial species [15, 19–21]. However, in real medical environments, the bacterial community consists of various bacteria originating from soil and skin surfaces and other organic matter [22]. Consequently, this study aims to explore the applicability of the contact plate method for sampling fabric microbial contamination levels in real medical environments.

## Methods

### Study design

From June to July 2021, a total of 12 wards were randomly selected in the Obstetrics Department of West China Second University Hospital, Sichuan University. Two privacy curtains were sampled in each ward, and a total of 24 were collected. Bacterial colony counting and identification were conducted on the surfaces of the privacy curtains using contact plate method and swab method. Sampling was done on the first day, seventh day, fourteenth day, and twenty-eighth day after the curtains were cleaned and disinfected. Patients were admitted to every study curtain during the study period, with no vacant beds. Curtains were not removed or replaced during the study.

### Contact plate method

The contact plate method employs the TSAWLPZS contact plate produced by Guangdong Huankai Microbial Co., Ltd., China. The contact plate is added with chlorine-containing and iodine-containing disinfectant neutralizing agent, the main components of which include pancreatic cheese peptone, soybean papain hydrolysate, sodium chloride, AGAR, lecithin, Tween 80, histidine and sodium thiosulfate. The surface area of each contact plate is 25cm^2^, and four contact plates were used to sample each curtain. The total area sampled was 100 cm^2^. In the culture medium, the convex surface of the plate was pressed for 5–10 s onto the surface of the curtain, and then the plate was covered and sent for analysis. The contact plates were placed in an incubator with a constant temperature of 35 °C for 48 h to perform bacterial colony counting and identification. The sampling area of the curtain corresponds to the high-touch area, i.e., “foot of the bed,” ranging from 60 to 140 cm from the ground. Two samples were collected from the patient-facing side and two from the healthcare personnel-facing side of the curtain.

### Swab method

The swab method was performed using chlorine and iodine-containing disinfectant neutralization sterile sampling solution produced by Wenzhou Kangtai Biotechnique Co., Ltd., China. The main components are beef powder, peptone, sodium chloride, and sodium thiosulfate. Sampling methods refer to Regulation for Washing and Disinfection Technique of Medical Textiles in Healthcare Facilities (WS/T 508–2016), which issued by the National Health and Family Planning Commission of the People’s Republic of China [23]. In the adjacent area to the contact plate sampling site, a cotton swab soaked with a sterile sampling solution was used to horizontally and vertically swipe five times within a 5 cm × 5 cm sterile culture dish. After each swipe, the cotton swab was rotated, and four culture dish areas were continuously sampled. The total area sampled was 100 cm^2^. The cotton swab (tip cut-off) was inoculated into a test tube containing 9 ml of sterile sampling solution and sent for analysis. After thoroughly shaking the sampling tube, 1.0 ml of the sampling solution was inoculated onto a sterile nutrient agar culture medium. The culture dish was incubated at 35 °C for 48 h to count and identify bacterial colonies.

### Colony counts and pathogenic bacteria identification

The swab method calculates the average colony count of each curtain directly after culture, while the contact plate method calculates the total colony number in the four contact plates after incubation in the contact plate, and then divided by the sampling area to calculate the average colony number of each curtain. VITEK MS automatic rapid microbial mass spectrometry detection system was used for strain identification. After 48 h of culture by the two sampling methods, the appropriate amount of bacteria was selected from the medium and transferred to the corresponding target position on the target plate. After drying for 30 min, the substrate liquid was added to cover it, and then natural air drying to form co-crystallization, the target plate could be tested on the machine. In order to ensure the reliability of the clinical identification results, they are reviewed by a microbiological expert before the mass spectrometry identification system is formally reported.

### Statistical analysis

The bacterial load detected by two sampling methods at different time points was graphically represented using GraphPad Prism 9.0. The comparison of bacterial load and species differences between the two methods at different time points was analyzed using the linear mixed-effects model method in SPSS 23.0. The model analysis included the sampling method, sampling time, room type where the curtain was located, and curtain position as fixed covariates. The model calculation was performed using the restricted maximum likelihood method. Chi-square test was performed on enumeration data. All tests used a two-sided significance level, with α = 0.05, and differences were considered statistically significant when *P* < 0.05.

## Results

### Curtain distribution

A total of 24 privacy curtains were sampled for this study, with 62.50% of them from double-occupancy rooms and 37.50% from triple-occupancy rooms. Among these curtains, 37.50% were located near the window, 16.67% were in the middle, and 45.83% were near the door.

### Bacterial load detected by two sampling methods

**Fig. 1:**
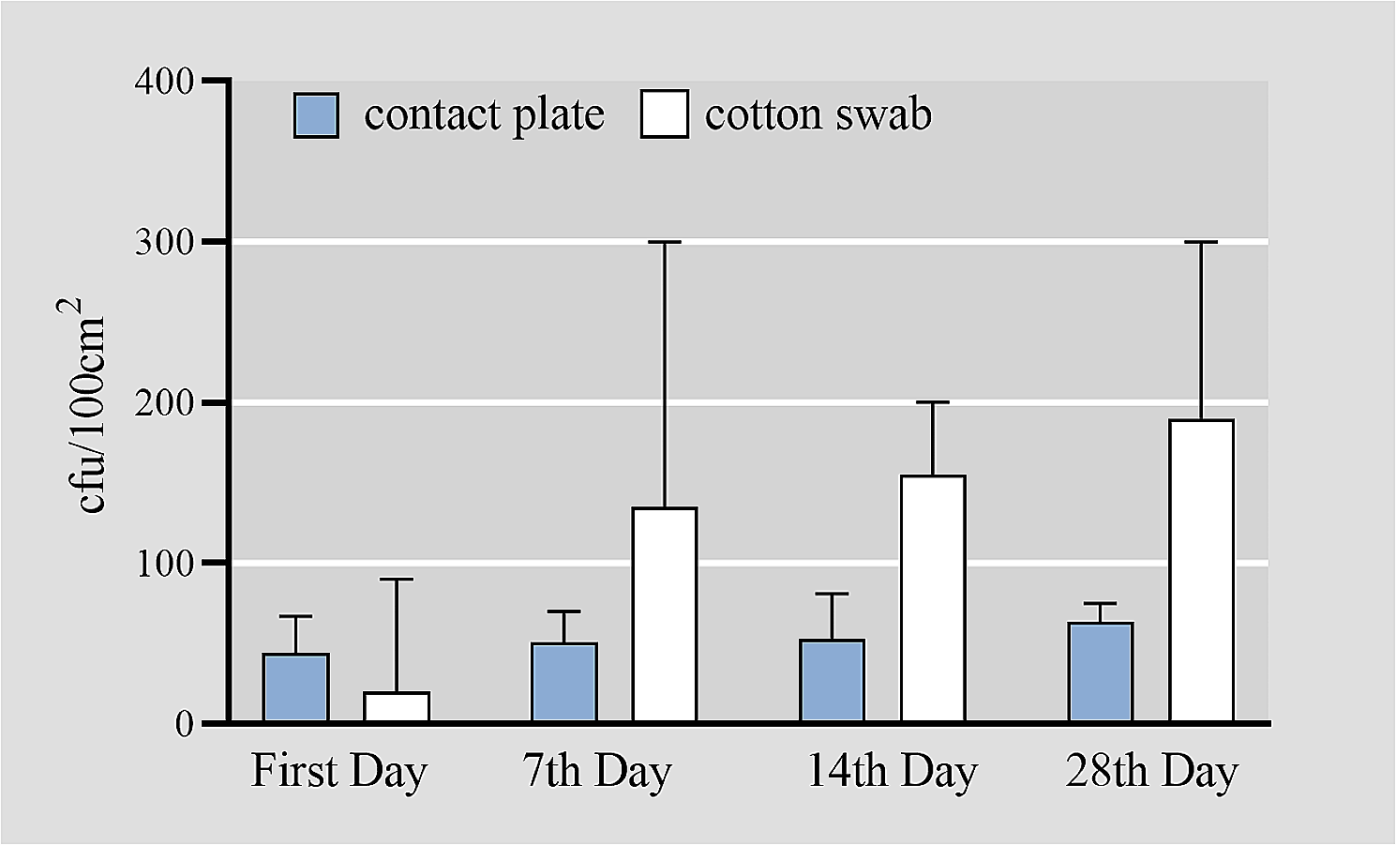
Bacterial load detected by the contact plate and cotton swab method at each time point(median with 95%CI)

The contact plate and cotton swab methods showed increased bacterial load on curtains with prolonged hanging time. Except on the first day, when the bacterial load detected by the contact plate method was greater than that detected by the cotton swab method, the bacterial load detected by the contact plate method was lower than that detected by the cotton swab method at the other sampling time points (Fig. 1). According to the mixed linear model analysis, the sampling method, sampling time, room type, and curtain location all impacted the bacterial load detected. The bacterial load detected by the contact plate method was lower than that of the cotton swab method (*P* < 0.001). Moreover, the bacterial load in double-occupancy rooms was lower than in triple-occupancy rooms (*P* = 0.021), and the bacterial load on curtains near the window was lower than those near the door area (*P* = 0.004, Table 1).

**Table 1:** The fixed effects of bacterial load estimated from the mixed linear model

Parameter	Estimate	Std. Error	t	*P*	95% CI	
					Lower Bound	Upper Bound
Intercept	271.46	30.81	8.81	<0.001	210.23	332.67
Contact plate = 1	-72.03	19.38	-3.72	<0.001	-110.62	-33.43
Contton swab = 2(control)	0	0				
First day = 1	-103.44	26.15	-3.96	<0.001	-155.58	-51.29
7th day = 2	-26.41	32.60	-0.81	0.419	-91.12	38.30
14th day = 3	-24.25	46.78	-0.52	0.606	-117.741	69.24
28th day = 4(control)	0	0				
Double-occupancy room = 1	-55.97	23.80	-2.35	0.021	-103.33	-8.61
Triple-occupancy room = 2(control)	0	0				
Near the window = 1	-64.62	21.79	-2.97	0.004	-108.01	-21.23
In the middle = 2	-44.86	31.25	-1.44	0.154	-107.02	17.31
Near the door = 3(control)	0	0				

### Detection of microorganisms using two sampling methods

After fitting a mixed linear model, it was observed that the fixed effects of the sampling method and sampling time influenced the number of isolated microbial species. The contact plate method yielded more isolated species than the cotton swab method (*P* < 0.001). Table 2 depicts that the number of detected species was lower on day 1 and day 14 (*P* = 0.001 and *P* < 0.001) compared to day 28. Table 3 presents that the predominant microbial species isolated by both methodologies were gram-positive bacteria. The detection rate of gram-positive bacteria by the contact plate method was lower than that by the cotton swab method, but there was no difference in the detection rate of gram-negative bacteria. Following the 28-day curtain microbial sampling, 8.33% of the curtains were contaminated with *S. aureus*, 12.50% with *A. baumannii*, 4.17% with *K. pneumoniae*, 4.17% with *P. aeruginosa* and 12.50% with Aspergillus, as shown in Table 4.

**Table 2:** The fixed effects of microbial species estimated from the mixed linear model

Parameter	Estimate	Std. Error	t	*P*	95% CI	
					Lower Bound	Upper Bound
Intercept	1.82	0.18	10.02	<0.001	1.46	2.19
Contact plate = 1	1.71	0.13	12.88	<0.001	1.45	1.98
Contton swab = 2(control)	0	0				
First day = 1	-0.56	0.17	-3.37	0.001	-0.89	-0.23
7th day = 2	-0.58	0.15	-3.84	<0.001	-0.88	-0.28
14th day = 3	0.06	0.15	0.42	0.677	-0.24	0.36
28th day = 4(control)	0	0				
Double-occupancy room = 1	-0.15	0.16	-0.91	0.366	-0.47	0.18
Triple-occupancy room = 2(control)	0	0				
Near the window = 1	-0.17	0.15	-1.16	0.251	-0.47	0.13
In the middle = 2	0.20	0.21	0.96	0.342	-0.22	0.62
Near the door = 3(control)	0	0				

**Table 3:** Distribution of microorganism using two sampling methods

	Contact plate			Contton swab		χ²	*P*
	*n*	%		*n*	%		
**Gram-positive bacteria**	**211**	**72.51**		**123**	**92.48**	**21.777**	**<0.001**
*M. luteus*	79	27.15		21	15.79		
*S. epidermidis*	52	17.87		53	39.85		
*S.hominis*	51	17.53		39	29.32		
*S. aureus*	3	1.03		2	1.50		
*Bacillus*	23	7.90		6	4.51		
*other staphylococcus*	3	1.03		2	1.50		
**Gram-negative bacteria**	**27**	**9.28**		**6**	**4.51**	**2.89**	**0.089**
*M.osloensis*	5	1.72		1	0.75		
*A. baumannii*	3	1.03		1	0.75		
*P. aeruginosa*	1	0.34		0	0.00		
*K. pneumoniae*	1	0.34		0	0.00		
*S. maltophilia*	0	0.00		1	0.75		
*other Gram-negative bacteria*	17	5.84		3	2.26		
**Mold**	**53**	**18.21**		**4**	**3.01**	**18.137**	**<0.001**
*Aspergillus*	3	1.03		0	0.00		
*other molds*	50	17.18		4	3.01		

**Table 4:** Frequency of microorganisms recovered from the privacy curtains

	*N*	Contact plate			Contton swab	
		*n*	%		*n*	%
**Gram-positive bacteria**						
*M. luteus*	24	24	100.00		16	66.67
*Bacillus*	24	18	75.00		6	25.00
*S. epidermidis*	24	16	66.67		21	87.50
*S.hominis*	24	16	66.67		14	58.33
*S. aureus*	24	2	8.33		2	8.33
*other staphylococcus*	24	3	12.50		2	8.33
**Gram-negative bacteria**						
*M.osloensis*	24	4	16.67		1	4.17
*A. baumannii*	24	3	12.50		1	4.17
*K. pneumoniae*	24	1	4.17		0	0.00
*P. aeruginosa*	24	1	4.17		0	0.00
*S. maltophilia*	24	0	0.00		1	4.17
*other Gram-negative bacterias*	24	16	66.67		3	12.50
**Mold**						
*Aspergillus*	24	3	12.50		0	0.00
*other molds*	24	23	95.83		4	16.67

## Discussion

No large-scale epidemiological study has demonstrated a direct correlation between contaminated privacy curtains and hospital-acquired infections [24]. However, reports in the scientific literature link them to hospital infection outbreaks [12, 13]. Privacy Curtains in prolonged contact with patients will likely become contaminated with blood and body fluids, posing potential pathogen contamination [25]. Previous research showed that 22% of clinical environment curtains are contaminated with MRSA, and 42% are contaminated with VRE [26]. *S. aureus* is the second most common pathogen causing HAIs [11]. Other studies indicated that hospital-related bacteria (*P. aeruginosa, Escherichia coli, and A. baumannii*) could survive on fabric for over a month, with moisture extending their survival time [15]. Conversely, compliance with hand hygiene after contacting the patient’s surroundings is relatively low among healthcare workers during the five key moments of hand hygiene [27]. Consequently, they are more likely to transfer microorganisms from the curtains to the patients, leading to HAIs.

Researchers showed that contact plates are more effective in laboratory contexts than cotton swabs at recovering *S. aureus* or MRSA from stainless steel surfaces and fabric surfaces [18, 20, 28, 29]. Lemmen et al. demonstrated that contact plates have higher sensitivity in detecting gram-positive cocci [17]. However, Okamoto et al. found that broth enrichment nylon swabs yielded more MRSA and VRE than contact plates to recover multidrug-resistant organisms in ICU wards [30]. Lerner et al. also found that the contact plate method had a lower detection rate than the swab method when detecting CRE on irregular and uneven surfaces like fabric [31]. Differences in the composition of organic matter, other microorganisms, and residues of cleaning or disinfecting agents on the surfaces of real healthcare environments may contribute to different research results. The present study showed that, after excluding the effects of room types and curtain positions on bacterial detection, the contact plate sampling method yielded lower bacterial counts than the cotton swab technique, consistent with the findings of Erikson et al. [32].

This study used two sampling methods to detect differences in bacterial counts, which may be attributed to two main reasons that differ from previous research. (1) Variation in the surface structure of the sampled objects: Contact plates revealed higher recovery efficiency on smooth, non-porous surfaces than cotton swabs in a study comparing the microbial recovery efficiency on different surfaces [18]. In contrast, textile materials are woven from yarns, resulting in rough and uneven surfaces, which makes it challenging to capture microorganisms within the three-dimensional structure of the fabric, leading to lower microbial detection rates. (2) Differences in material properties: Rabuza et al. found that contact plates performed better than cotton swabs in recovering microorganisms from 100% cotton fabric [15]. However, in this study, the target material was composed of 100% polyester fibers. Considerable evidence suggests that the surface material of textiles is also one of the factors influencing sampling efficiency. According to existing literature reports, microorganisms exhibit stronger adhesion to polyester fibers than to 100% cotton, resulting in greater biological recovery rates for cotton materials [29]. Nonetheless, this study requires further in-depth research, which could be explored in future investigations.

The present study revealed that the bacterial counts detected on the curtain in double-occupancy rooms were lower than in triple-occupancy rooms, possibly due to the lower occupancy and reduced frequency of curtain touch in double-occupancy rooms, leading to lower contamination probability. Additionally, bacterial counts on the curtain were lower near the window than near the door. This observation could be attributed to the fact that curtains near the window are on the innermost side of the room, with less contact from other individuals. Furthermore, except for the first day of cotton swab sampling on the curtain, which showed lower counts than contact plates, all other occurrences of bacterial counts found with cotton swabs showed greater counts than contact plates. This outcome might be related to varying levels of microbial contamination on the curtain, as some studies indicate that cotton swabs perform better in recovering microorganisms in situations with high surface contamination levels, whereas contact plates perform better when the contamination is low [15].

The results of this study showed that the number of bacterial species detected using the contact plate method was higher than that of the cotton swab method. Most of the microbiota identified by both sampling methods were skin and environmental bacteria. Gram-positive bacteria (72.51%) were the most prevalent, followed by fungi (18.21%), which is consistent with the findings by Woodard and colleagues in the emergency department [33]. Notably, 12.5% of the curtains in this study were contaminated with molds, which should alert infection control personnel, particularly in wards with immunocompromised patients, such as oncology and burn units. Since fungi primarily spread through spores, any curtain is susceptible to colonization. Previous studies reported nosocomial outbreaks of mold infections caused by exposure to airborne molds during hospital construction, particularly in patients undergoing hematopoietic stem cell transplantation [34]. Furthermore, mold infections are a significant cause of mortality in severely immunocompromised patients, with 50% of deaths in hematologic malignancies, hematopoietic stem cell transplant recipients, and severe immunodeficiency patients associated with mold infections [34]. In this study, only 8.33% of the curtains were contaminated with *S. aureus*, and 12.50% with *A. baumannii*, which was lower than the results of reported by Ohl and Kevin in a burn/plastic ward, possibly due to differences in patient populations in the respective wards [35, 36].

### Limitations and strengths

A limitation of this study is that both sampling methods were performed at adjacent locations, and bacterial distribution on the curtains may be uneven, potentially affecting the research results. At the same time, cotton swab method has the problem of bacterial load limit.

## Conclusion

In summary, different sampling methods can be selected according to the monitoring purpose. If the purpose is to understand the species of contaminated bacteria on fabrics, the contact plate method is recommended. While if the purpose is to assess the level of curtain contamination, the cotton swab method is more suitable than contact plate.

## Data Availability

The datasets analysed during the current study are available from the corresponding author on reasonable request.

## Abbreviations

A. Baumannii: Acinetobacter baumannii
HAI: Hospital-acquired infection
HAIs: Hospital-acquired infections
ICU: Intensive care unit
K. Pneumoniae: Klebsiella pneumoniae
M. luteus: Micrococcus luteus
MRSA: Methicillin-resistant Staphylococcus aureus
P. aeruginosa: Pseudomonas aeruginosa
S. Aureus: Staphylococcus aureus
S. epidermidis: Staphylococcus epidermidis
S. hominis: Staphylococcus hominis
M. osloensis: Moraxella osloensis
S. maltophilia: Stenotrophomonas maltophilia
VRE: Vancomycinresistant Enterococcus
